# 

**Published:** 1889-05

**Authors:** 


					﻿Vol. IX.
MAY, 1889.
NO. 5.
THE
pOMEOPATHlC PHYSICIAN,
A Monthly Journal of Homoeopathic Materia Medica
and Clinical Medicine.
“ He is a freeman whom truth makes free, and all are slaves beside.”,
"Seek the Truth: come whence it may, cost what it will.”
EDITED AND PUBLISHED BY
Edmund j. lee, ml d.,
AND
WALTER M. .JAMES, ML E>.
PHILADELPHIA : «
No. lies SPRUCE STREET.
LONDON
ALFRED HEATH & CO., Sole Agents fob Great Britain,
114 Ebury Street, Eaton Square.
SS* Yearly Subscription, $2.50. invariably in advance. Single numbers, 25 cts.
Single numbers containing the Repertory Supplement, 50 cts.
Entered at the Philadelphia Poet-Office as Second-Class Mail Matter.
				

